# Molecular docking analysis of aplysin analogs targeting survivin protein

**DOI:** 10.6026/97320630013293

**Published:** 2017-09-30

**Authors:** Eram Shakeel, Salman Akhtar, Mohd. Kalim Ahmad Khan, Mohtashim Lohani, Jamal M. Arif, Mohd. Haris Siddiqui

**Affiliations:** 1 Advanced Centre for Bioengineering and Bioinformatics (ACBB), Integral Information and Research Centre (IIRC), Integral University, Lucknow, Uttar Pradesh, India-226026; 2 Department of Bioengineering, Faculty of Engineering, Integral University, Lucknow, Uttar Pradesh, India-226026; 3 Department of Biosciences, Integral University, Lucknow, Uttar Pradesh, India-226026

**Keywords:** Marine, Apoptosis, Survivin, Aplysin, analogs

## Abstract

Survivin (IAP proteins) remains an important target for anticancer drug development as it is reported to be over-expressed in tumor cells to enhance resistance to apoptotic stimuli. The study focuses on virtual screening of marine compounds inhibiting survivin, a multifunctional protein, using a computational approach. Structures of compounds were prepared using ChemDraw Ultra 10. Software and converted into its 3D PDB structure and its energy was minimized using Discovery Studio client 2.5. The target protein, survivin was retrieved from RCSB PDB. Lipinski's rule and ADMET toxicity profiling was carried out on marine compounds and the filtered compounds were further promoted for molecular docking analysis and interaction studies using AutoDock Tools 4.0. Molecular docking results revealed that analog (AP 4) of Aplysin, showed very promising inhibitory potential against survivin with a binding energy of -8.75 kcal/mol and Ki 388.28 nM as compared to its known inhibitor, Celecoxib having binding energy of -6.65 kcal/mol and Ki 13.43 μM. AP 4. The analog depicted similarity in pattern when compared to standard. The result proposes AP 4, is an effective molecule exhibiting prominent potential to inhibit survivin and thus promoting apoptosis in tumor cells.

## Background

Apoptosis being an important mechanism for inhibiting cancer progression is well reported to be targeted by researchers for the development of chemotherapeutic agents. Marine natural products have been a fascinating area of research towards development of anticancer drugs. Sesquiterpenoids of marine origin is reported to possess strong anticancer potential by inhibiting cell proliferation or through cell death induction [1]. Undoubtedly, restraining apoptosis is becoming a hallmark in several cases of cancer as reported [2]. Depending on this idea, augmented levels of diverse members of the IAP family have been reported in many cancer types [3] and over-expression of IAP proteins has been reported to enhance resistance to apoptotic stimuli in many malignancies [4]. The IAPs (Inhibitor of Apoptosis) are reported as conserved during the evolutionary process, both in vertebrate and invertebrate animal species [3]. Survivin (BIRC5) (baculoviral IAP repeat contacting 5) being an important member of the inhibitor of apoptosis (IAP) family is known to be associated in both cell survival and the regulation of mitosis in tumor cells [5]. Survivin is the smallest family member of IAPs comprised of 142-amino acid of 16.5 kDa encoded by a single gene located on the human 17q25 chromosome, consisting of three introns, and four exons [6] and exists physiologically as a functional homodimer [7]. The report suggests that expression of survivin occurs in embryonic tissues and most tumor tissues, but not in normal mature tissues. The highly selective nature of survivin expression makes it an important prognostic marker, for inducing apoptosis in oncogenic cells [8] by blocking caspase activation. Abnormally towering expression of survivin is associated with multiple cellular processes like tumor cell proliferation, progression, angiogenesis, therapeutic resistance, and poor prognosis [9]. Previously it was accounted that survivin restrains cell death persuaded via the extrinsic and intrinsic apoptotic pathways and bestows resistance to apoptosis by directly repressing caspase activity. A current report suggests that survivin functions upstream of the effector caspases, by inhibiting caspase 9 through the formation of a survivin-hepatitis B X-interacting protein (HBXIP) complex bound to pro-caspase-9, thus averting the recruitment of apoptotic protease activating factor 1 (Apaf-1) to the apoptosome [10]. Facts suggests that survivin is also associated with the up-regulation phosphatidylinositol 3-kinase (PI3K)/Akt pathway resulting in cell survival and resistance to apoptosis in different malignant cells, including myeloid leukemia and cancers of prostate, breast and lung [11]. Survivin is also well reported to be responsible for up-regulating vascular endothelial growth factor (VEGF) and inducing angiogenesis in tumors by accumulating β-catenin in the cytoplasm and inducing its translocation to the nucleus to form then β-catenin/T-cell factor (TCF) transcriptional activator that up regulates VEGF [9].

In one of the mechanism, Cdc2 gets phosphorylated, which in turn phosphorylates survivin allowing it to form complex with Cdk1, thus arresting the cell cycle in G2M phase causing uncontrolled mitosis.Survivin plays a central role in inducing cell division viaextrinsic and intrinsic apoptotic pathways. Suppression of Caspase activity via survivin inhibition bestows resistance to apoptosis in cancer cells. As a new reported mechanism, Survivin works by inhibiting Caspase 9 in an upstream manner [10]. It is well reported about survivin that it inhibits apoptosis both in vitro and in vivo [12] by interacting with several regulators of intrinsic and extrinsic pathways of apoptosis. Survivin is known to inhibit apoptosis both in vitro and in vivo perhaps via interactions with multiple regulators of both intrinsic and extrinsic apoptosis pathways. Survivin is negatively regulated by p53, both at the mRNA and protein [13].

In addition to its role in suppressing apoptosis, survivin is also a mitotic regulator involved in various cell division processes via localization at the mitotic apparatus. The microtubule-assembled survivin upon association with CDK1 becomes phosphorylated stabilizing survivin during mitosis and thus repressing cell death in mitotic cells. Survivin is a key constituent of the chromosomal passenger complex (CPC) and thereby functions as a key regulator of chromosomal segregation and cytokinesis. In addition, it has been well studied that activation of the checkpoint kinase 2 (CHK2) due to DNA damage outcomes in rapid release of survivin from the mitochondria and therefore inhibiting cell death that upholds tumor cell survival. Due to its role in many different cellular actions and signaling pathways, survivin has been described as a nodal protein. Survivin has been reported as well expressed in different cancers such as glioblastoma, lung cancer, hepatocellular carcinoma, B-cell non- Hodgkin's lymphoma, esophageal cancer and breast cancer patients causing reduced survival rates [14].

**Figure 1:**
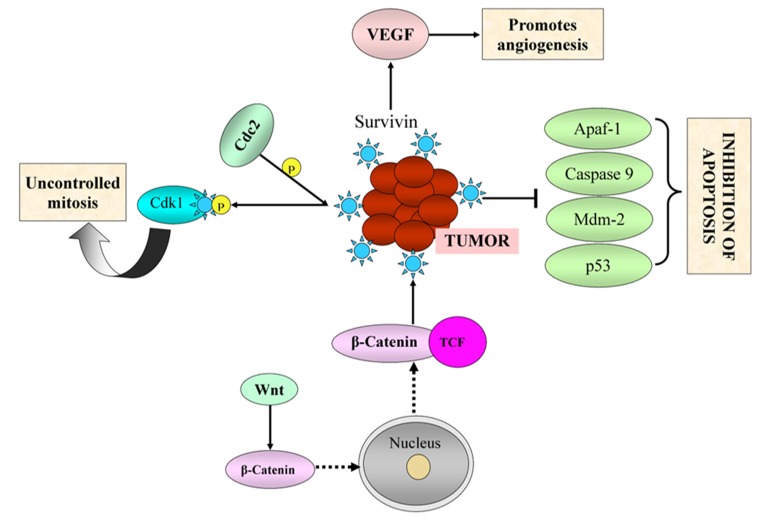
Mechanism of Survivin Pathway: Overexpression of survivin promotes tumor cell survival through (i) inactivation of Apaf-1, Casapase 9, Mdm-2, p53 (ii) causes uncontrolled mitosis in tumor cells through Cdk1 (iii) promotes angiogenesis through activation of Vascular endothelial growth factor (VEGF).

It is also stated through various in vitro and in vivo studies that survivin also inhibits Apaf-1 and Mdm-2 protein inhibiting apoptosis in cancer cells. These promising reports suggest that survivin could serve as a marker for the diagnosis of malignancies at early stages [14]. Thus, targeting survivin for cancer therapy can be a novel approach as it is involved in multiple signaling pathways. The overall mechanism of survivin pathway is depicted in Figure 1.

Aplysin (C15H19BrO), a bromo sesquiterpene compound isolated from Laurencia tristicha (seaweed) with a molecular weight of 295 is reported to reduce ethanol-induced hepatic injury in mice [15] and it also sensitizes cancer cells to TRAIL by suppressing P38 MAPK/Survivin pathway [16]. However, its probable appliance for anti-cancer therapy has not been yet explored.

Celecoxib, a selective cyclooxygenase-2 (COX-2) inhibitor, is the only FDA (Food and Drug Administration) approved drug for the treatment of FAP (Familial adenomatous polyposis) patients that is known to induce apoptosis and suppressed the survivin expression in HCT-116 cells [17].

Therefore, the study has been used to discover the anticancer potential of aplysin targeting survivin. The study has been carried out successfully to predict the role of marine compound Aplysin and its designed novel analogs as anti-survivin agent, thereby inducing apoptosis in cancer cells.

## Methodology

### Retrieval of 3-D Structure of target Survivin

The 3-D crystal structure of protein was extracted from RCSB protein Databank using X-ray diffraction studies with resolution of 2.55 Å (PDB ID: 1F3H) [18]. Finally energy minimization of the constructed structure was performed using CHARMm forcefield and MMFF94x partial charge and further minimized using RMS gradient energy with 0.001 kcal/mol keeping all the other parameter at the default.

### Preparation of Compounds

The 2D-structure of Aplysin and its 50 analogs were constructed using ACD lab software extension ChemDraw in MDL .mol format and was imported to Discovery Studio 2.5 window for generation of 3D-structure. The 3D structure was optimized using CHARMm forcefield and MMFF94x partial charge and further minimized using RMS gradient energy with 0.001 kcal/mol keeping all the other parameter at default (Table 1).

### Drug-likeliness Prediction of Aplysin Analogs

To estimate solubility and permeability of compounds by using computational approaches, 'the rule of 5's is applied. This rule estimates the pharmacological, biological and ADME (absorption, distribution, metabolism and excretion) activity of the particular compound thus, predicting its potential as an orally active drug in humans [19].

The 'rule of 5' states that: poor absorption or permeation is more likely when:

a) There are more than 5 H-bond donors [expressed as the sum of OHs and NHs) b) The molecular weight is over 500 c) The Log P [octanol-water partition coefficient) is over 5 d) There are more than 10 H-bond acceptors [expressed as the sum of Ns and Os) e) Compound classes that are substrates for biological transporters are exceptions to the rule.

### ADMET prediction of Aplysin Analogs

Using PreADMET online server (http://preadmet.bmdrc.org) the pharmacokinetics parameters like Adsorption, Distribution, Metabolism, Excretion and Toxicology (ADME/T) was calculated. This aspect calculates the property like Human Intestinal Absorption (% HIA), Caco-2 permeability, MDCK cell Permeability, Skin Permeability, Blood Brain Barrier Penetration and Carcinogenicity. Compounds filtered through Lipinski were sorted out on the basis of ADMET properties for the further Docking simulation process. The standard range of ADME parameters are given in the Table 4.

### Docking Simulation

The virtual screening was performed using the Auto-dock Tool 4.0 [20] to find preferred binding conformation of ligand and receptor. Binding conformation of protein-ligand complex was analyzed using a scoring function of free energy of binding [21]. Autodock recommended Lamarckian Genetic Algorithm (LGA) to determine globally optimized conformation to determine globally optimized conformation. Using Autodock tools polar hydrogen atom, Kollman charges, atomic solvation parameters and fragmental volume were allocated to protein. The grid spacing was 0.375Å between 2 connecting grid points. Each grid point in x, y, z axes was 60 x 60 x 60Å and X=29.127, Y= 1.747, Z= 3.891co-ordinates. In every docking test, 25 runs were executed and population size was set at 150, maximum number of evaluation was 2,500,000, maximum number of generation was 27,000, rate of gene mutation 0.02 and cross-over rate 0.8. Rest parameter was set to default. For each docking experiment RMSD (Root Mean Square Deviation) was set to 2.0Å. Inhibitor molecule constitutes 0.274 coefficients of tensional degree of freedom. After completion of ligand-protein complex was achieved. The final was decided on the basis of interaction energy and inhibition constant (Ki).

## Results

### Drug-Likeliness Prediction of Analogs

Differentiate between "drug-like" compounds from non-drug like compound is a key spotlight on recent research in computer aided drug designing field. Technique for drug-likeliness investigation consists of Lipinski's rule of 5. If the violation is 1 or 0 it comprises that compound easily bind to receptor [22]. If the violation number exceeded than 2, compound was rejected from further selection [23]. Out of 50 Aplysin analogs, only 38 analogs were filtered through Lipinski's rule of 5 and were passed for its ADMET prediction. Filtered analogs are listed below (Table 2).

### ADMET study of novel Analogs

Traditionally, testing compounds is time-consuming multistep processes. Potential compounds then further investigated for development where their pharmacokinetics properties, metabolism and potential toxicity were examined. Therefore, combinatorial chemistry and high throughput ADME screens were now employed. ADMET prediction of analogs was completed by online tool PreADMET (preadmet.bmdrc.org). Out of 38 filtered analogs, 22 analogs were selectedfor further docking studies (Table 3).

### Docking Simulations of novel analogsand standard Drug with Survivin

For understanding the structural basis of protein-ligand specificity docking approach was used. Docking studies were conducted on analogs selecting after in silico filter (ADMET and Lipinski's Rule) against Bcl-2 target. The compound was selected on the basis of Binding energy and Inhibition Constant (Table 4).

## Discussion

Various marine compounds known for its anticancer activity have been discontinued for further clinical trial due to rigorous side effects imposed by them. Due to its severe toxicity profile Didemnin B, marine natural compound isolated from Trididemnum solidum was removed from further clinical trial [24]. Another compound Cematodin also imposed severe side effects, including cardiac toxicity, hypertension, and acute myocardial infarction and the most common effect of neutropenia leading to its discontinuation in clinical assessment [25]. Dolastatin 15 has also been removed from a clinical trial in preclinical studies. Regrettably Dolastatin 10 was taken away from clinical trials owing to the development of moderate peripheral neuropathy in 40% of patients and insignificant activity in patients with hormone refractory metastatic adenocarcinoma and persistent platinum-sensitive ovarian carcinoma [24].

Apoptosis inhibition is a hallmark phenomenon in the majority of cancer cases. In this respect different members of IAP proteins have been reported as overexpressed in cancer cells, thus responsible for inhibiting the apoptotic process within them. Particularly survivin, a nodal protein of this family has gained a crucial attention due to its remarkable specific expression in cancer cells [14]. Considerable efforts have been focused on developing approaches to use survivin as a target for therapeutics in cancer. Survivin is not an enzyme nor is it a surface protein. Thus, targeting it might be difficult for drug development. However, significant advancement has been made to attain optimal efficiency in suppressing survivin.

Present study conducted on Aplysin, a marine compound and its analogs revealed an interesting fact that Aplysin, a sesquiterpene compound possesses potent anticancer property against survivin based on the binding energy produced during docking analysis. Its analog also exhibits a better anticancer profile as compared to its parent compound aplysin.

Docking study of Aplysin and its analogs against the target protein survivin showed that AP4, AP11 and AP29 showed the best binding energy among all other analogs compared to the standard Celecoxib. The binding energies of AP4, AP11 and AP29 came out to be -8.75 kcal/mol, -8.72 kcal/mol and -8.02 kcal/mol respectively that was much greater than the standard compound Celecoxib (-6.65 kcal/mol). The complex of AP 4 and survivin was found to be more stable due to the formation of five hydrogen bonds as compared to AP11 and AP 29 that showed only two and three hydrogen bonds respectively. When compared to their Lipinski's Rule of five and ADMET properties like BBB, CaCO-2, HIA, PPB, SP and M/C, AP4 was found to be in harmony with the standard drug Celecoxib. Thus, on the basis of results obtained via computational studies of analogs, AP4 came out to be the best compound showing promising results targeting survivin and thus capable of inducing apoptosis in tumor.

The analog (AP 4) exhibited no violation as compared to its parent compound, Aplysin that followed all the parameters of Lipinski's Rule with one violation that is acceptable according to standard rule of five. The comparative study of analogue AP 4 with standard drug Celecoxiband the parent compound Aplysin, showed that it surmounts the various ADMET properties like BBB, CaCO-2, HIA, PPB, SPand M/C properties as discussed in Table 3, but the major point to judge it as a lead compound is that it is a non mutagen and non-Carcinogen both in case of rat and mouse as compared to standard drug Celecoxib and parent compound Aplysin as per PreADMET study. As the standard drug (FDA approved) was found to be mutagenic Thus, Aplysin and its analogs were taken forward for its interaction study with target protein BCl-2.

**Figure 2:**
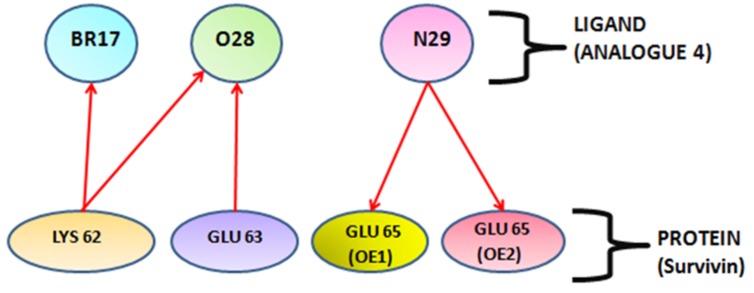
H-Bonding interaction of the ligand (analog 4) and Survivin protein

Plasma protein binding of analog 4 was found to be within the PreADMET standard (<90%) as compared to the parent compound (Aplysin) and the standard drug (Celecoxib) as mentioned in Table 3. As it is well reported that the amount of unbound drug in plasma is only responsible for showing effective pharmacological properties by binding to its target receptor in the tissues. Therefore, the PPB should be below 90 according to the PreADMET standards for showing enhanced activity when bound to its receptor [26]. Analogue 4 form five hydrogen bonds with K62, E63 and E65 respectively. The first and second hydrogen bonds were formed between K62 with Br 17 and O28 having 2.477Å and 2.29Å bond distance in which K act as donor and ligand with Br and O28 acting as an acceptor group. The third hydrogen bond was formed between the E63 amino acid of protein and O28 of ligand, having bond distance 2.299Å in which E63 act as donor and O28 act as acceptor. In remaining two hydrogen bonds, ligand acts as hydrogen bond donor and amino acid residues as H-bond acceptor. N29 position of ligand forms 2 hydrogen bonds with E65 amino acid at OE1 and OE2 position of protein having bond distance 3.09Å and 3.18Å respectively. The H-bond interaction between ligand (analogue 4) and protein (survivin) is illustrated in Figure 2. This hydrogen bond enhances the stability of analogue with the closely associated amino acid of protein. Hydrogen bonds between protein and ligand were found to be energetically significant as all the bonds formed linking donors and acceptor atoms are within 3.5 Å of each other.

**Figure 3:**
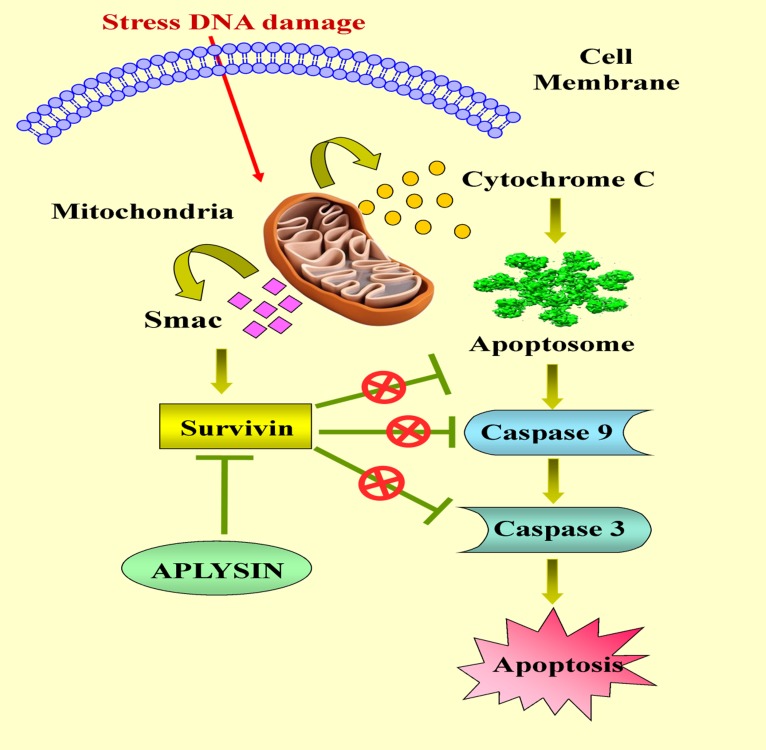
Anti-apoptotic effect of Aplysin inhibit Survivin protein to induce apoptosis which acting at different points in Apoptotic Pathway

Thus, it is predictable that Aplysin and its analog (AP 4) inhibit survivin exerting anti apoptotic effect. It is also thus proposed that Aplysin and its analog (AP 4) has increased retention time in the body with enhanced medicinal effect on survivin over expressing tumor cells. Targeting survivin has become a strong rationale for antitumor drug development due to its differential expression of survivin in malignant versus normal cells. Survivin is well reported to be upregulated in a variety of human cancers exhibiting a further belligerent phenotype, shorter survival times, and a decreased response to chemotherapy [27]. Accordingly, antisurvivin therapy would be a novel approach for overcoming the decreased chemotherapeutic response in cancer patients. The Figure 3 represents the possible consequences of inhibiting survivin. Stress induced DNA damage causes a rapid release of survivin from mitochondria, inhibiting cell death and promoting tumor cell survival by inactivation of apoptosome and promotion of caspase cleavage.

## Conclusion

The chronological results of various kinds of marine compounds in existence are associated with novel antitumor drugs exhibiting pioneering treatments for malignancy in the future Promoting cell death is the essential ground in terms of anticancer therapy. On the basis of studies, it can thus be concluded that Analog 4 (AP4) of Aplysin (marine originated compound) can prove to be a compelling remedial agent against survivin, capable of promoting apoptosis pathway by inducing cell death. Docking studies carried out on Aplysin and its analog AP4 against standard drugs, Celecoxib, showed better binding energy revealing the fact that it may have the potential to inhibit survivin (an important prognostic marker in cancer therapy) and possibly will come forward as striking marine compounds that induces cell death with lesser toxicity profiling.

## Conflict of interest

The authors confirm that this article content has no conflict of interest.

## Figures and Tables

**Table 1:** Pharmacokinetic parameters for the measurement of drug concentrations in blood or plasma.

S. No	ADME Properties	Activity range
1	Human intestinal absorption (HIA) absorption	Poorly- 0~20%
		Moderate- 20~70%
		High- 70~100%
2	Blood brain barrier (BBB)	CNS active compounds (+); >1
		CNS inactive compounds (-); < 1
3	Plasma protein Binding (% PBP)	Chemicals strongly bound > 90%
		Chemicals weakly bound < 90%
4	Madin-Darby canine kidney (MDCK) cell permeability	Lower- < 25
		Moderate- 25~500
		Higher- > 500
5	Heterogenous human epithelial colorectal adenocarcinoma (Caco2) cell permeability	Lower- < 4
		Moderate- 4~70
		Higher- < 70

**Table 2:** Structure of designed novel analogs

S.No.	Analogs	R1
1.	AP 1	-
2.	AP 4	-
3.	AP 10	Cl
4.	AP 11	-
5.	AP 12	Cl
6.	AP 13	Br
7.	AP 14	Br
8.	AP 15	Br
9.	AP 16	-
10.	AP 18	-
11.	AP 20	-
12.	AP 21	-
13.	AP 23	-
14.	AP 24	-
15.	AP 26	-
16.	AP 28	-
17.	AP 29	-
18.	AP 36	-
19.	AP 37	-
20.	AP 40	-
21.	AP 43	-
22.	AP 47	-

**Table 3:** Novel Analogs following Drug-Likeliness Test along with the standard drug

S. no	Analogs	Mol.Wt	NoN	NoHNH	Log P
	Celecoxib	381.373	3.4	1	7
	Aplysin	295.22	1	0	5.15
1	AP 1	236.74	1	0	4.26
2	AP 2	251.76	2	2	4.05
3	AP 3	284.32	1	0	5.25
4	AP 4	266.77	2	0	4.62
5	AP 5	265.78	2	1	4.42
6	AP 6	251.76	2	2	4.15
7	AP 7	253.73	3	3	2.76
8	AP 8	331.82	2	2	5.63
9	AP 9	350.26	1	1	6.53
10	AP 10	335.26	4	3	3.07
11	AP 11	477.85	2	0	6.77
12	AP 12	319.88	2	0	4.77
13	AP 13	384.75	2	0	4.75
14	AP 14	386.72	3	0	4.49
15	AP 15	385.72	3	1	3.94
16	AP 16	423.8	1	0	6.84
17	AP 17	400.74	3	0	4.87
18	AP 18	379.35	1	0	6.76
19	AP 19	388.36	3	0	5.08
20	AP 20	374	2	0	5.98
21	AP 21	409.04	3	0	5.81
22	AP 22	409.04	3	0	5.81
23	AP 23	444.22	1	0	7.14
24	AP 24	452.85	2	0	6.57
25	AP 25	425.77	2	1	6.25
26	AP 26	466.83	3	1	6.19
27	AP 28	466.83	3	1	6.19
28	AP 29	491.92	1	0	8.33
29	AP 34	463.82	2	1	7.44
30	AP 35	450.79	3	0	6.26
31	AP 36	455.81	4	1	5.07
32	AP 37	476.84	2	1	2.96
33	AP 38	497.86	3	0	6.95
34	AP 39	453.79	3	1	6.04
35	AP 40	451.81	2	0	6.23
36	AP 42	454.77	4	0	4.42
37	AP 43	486..77	6	0	5.3
38	AP 47	495.87	3	0	7.48

Abbreviations: Log P- partition coefficient; MW- molecular weight; nON-hydrogen bond acceptors; nOHNH- hydrogen bond donor.					

**Table 4:** ADMET profiling of novel marine Analogs with their parent compound

S.No.	Analogs	BBB	Caco2	PPB	HIA	SP	Toxicity (M/C)
Celecoxib		0.0272	0.499	91.07	96.68	91.07	+/--
Aplysin		6.3979	55.769	91.48	100	-1.153	+/--
1	AP1	6.051	56.37	91.07	100	-1.271	+/--
2	AP4	0.501	52.898	86.22	97.655	-2.474	-/--
3	AP10	1.011	0.532	93.37	94.491	-3.052	+/--
4	AP11	0.991	32.419	100	99.408	-2.078	+/--
5	AP12	4.6	57.01	85.24	100	-2.639	+/--
6	AP13	9.444	56.814	85.24	100	-2.792	+/--
7	AP14	1.506	56.068	96.39	100	-3.507	+/--
8	AP15	5.003	52.956	82.44	98.35	-3.599	+/--
9	AP16	0.286	57.61	96.85	99.244	-1.145	-/--
10	AP18	2.141	57.95	99.511	99.234	-1.164	+/--
11	AP20	2.033	57.16	93.35	98.525	-1.918	-/--
12	AP21	1.581	55.904	91.66	98.117	-3.054	-/--
13	AP23	2.628	57.71	99.22	99.218	-1.214	-/--
14	AP24	4.643	57.697	93.2	98.742	-1.647	-/--
15	AP26	3.423	55.039	93.84	97.302	-3.07	-/--
16	AP28	12.66	57.359	88.5	99.202	-0.847	-/--
17	AP29	0.259	2.304	94.56	97.193	-2.791	+/--
18	AP36	1.15793	50.0446	75.56	93.456	-3.9018	-/--
19	AP37	3.85254	47.4872	98.36	97.4917	-2.313	-/--
20	AP40	1.7326	56.98	97.58	98.244	-2.604	+/--
21	AP43	3.77079	1.58846	90.23	98.54	-1.006	+/--
22	AP47	1.24665	57.5445	97.25	98.08	-1.438	+/--

Abbreviations: BBB- Blood brain barrier; HIA-Human intestinal absorption; SP-Skin permeability; MDCK- Madin-Darby canine kidney; Caco-2- heterogenous human epithelial colorectal adenocarcinoma; M- mutagen; C-carcinogen(rat, mouse)							

**Table 5:** Binding energy of docked protein (Survivin) and novel Anticancer Analogs along with the standard Marine Compound Aplysin

S.No.	Analogs	Binding Energy (kcal/mol)	Inhibition Constant uM	No. of Hydrogen Bond	Residue Interaction
1	Celecoxib	-6.65	13.43	5	K62:HZ1 -: UNK0: N8
					K115:HZ3 -: UNK0: O5
					K115:HZ3 -: UNK0: O6
					UNK0: H39 - E63: OE2
					UNK0: H40 - E63: OE2
2	Aplysin	-5.83	53.04	0	No Hydrogen Bond
3	AP 1	-6.31	23.75	0	No Hydrogen Bond
4	AP 4	-8.75	388.28 nM	5	K 62:HZ1 -: UNK0: BR17
					K62:HZ2 -: UNK0: O28
					E 63:HN -: UNK0: O28
					UNK0: N29 - E 65: OE1
					UNK0: N29 - E 65:OE2
5	AP 10	-7.12	6.01	2	N111:HD22 -: UNK0:O18
					UNK0: N20 - A: G83:O
6	AP 11	-8.02	1.31	2	K62:HZ2 -: UNK0:O28
					E63: HN -: UNK0:O28
7	AP 12	-6.42	19.84	0	No Hydrogen Bond Form
8	AP 13	-6.03	37.98	0	No Hydrogen Bond Form
9	AP 14	-6.04	37.38	1	K122:HZ3 -: UNK0:O20
10	AP 15	-5.49	94.94	0	No Hydrogen Bond Form
11	AP 16	-6.92	8.43	0	No Hydrogen Bond Form
12	AP 18	-6.76	11.07	0	No Hydrogen Bond Form
13	AP 20	-6.02	38.57	0	No Hydrogen Bond Form
14	AP 21	-5.22	148.2	0	No Hydrogen Bond Form
15	AP 23	-7.3	13.73	1	S81: HG -: UNK0: BR17
16	AP 24	-6.76	11.08	1	K122:HZ3 -: UNK0: CL15
17	AP 26	-6.97	7.83	0	No Hydrogen Bond Form
18	AP 28	-7.01	7.24	1	K122:HZ3 -: UNK0:O9
19	AP 29	-8.72	407.25nM	3	K115:HZ3 -: UNK0:O30
					E63: HN -: UNK0:O29
					K62:HZ2 -: UNK0:O29
20	AP 36	-7.6	2.67	1	K115:HZ3 -: UNK0:O25
21	AP 37	-7.01	7.25	4	S81: HG -: UNK0:O29
					K122:HZ3 -: UNK0:O9
					UNK0:O29 - S81:OG
					UNK0:O29 - S81:O
22	AP 40	-6.97	7.76	2	A85: HN -: UNK0:O25
					N111:HD22 -: UNK0:O25
23	AP 43	-8.72	406.37nM	5	K62:HZ2 -: UNK0:O27
					E63: HN -: UNK0:O27
					K115:HZ3 -: UNK0:O28
					N119:HD22 -: UNK0:O25
					K122:HZ3 -: UNK0:O26
24	AP 47	-5.88	48.72	2	R108:HH11 -: UNK0:O24
					R108:HH11 -: UNK0:O25
